# From inflammatory effectors to tissue sculptors: non-canonical functions of neutrophils in repair, barrier remodelling and fibrosis

**DOI:** 10.3389/fimmu.2026.1891187

**Published:** 2026-07-01

**Authors:** Jiaqi Liu, Junzhe Chen, Ziyi Luo, Yuezhong Chen, Shaoxiang Yuan, Jin Li

**Affiliations:** 1 Key Laboratory of Infectious Diseases and Biosafety, Education Department of Guizhou Province, Zunyi Medical University, Zunyi, Guizhou, China; 2 Department of Burns and Plastic Surgery, Affiliated Hospital of Zunyi Medical University, Zunyi, Guizhou, China

**Keywords:** barrier, fibrosis, immunity, matrix, neutrophils

## Abstract

Traditionally, neutrophils have been viewed as short-lived effector cells of acute inflammation, specialized for chemotaxis, phagocytosis, degranulation, reactive oxygen species production and neutrophil extracellular trap formation. This view remains central to host defence, but emerging evidence indicates that, in defined tissue and disease contexts, neutrophils can also regulate tissue architecture. In this review, we explain how canonical neutrophil effector mechanisms acquire structural consequences, focusing on neutrophil–matrix interactions during wound repair, barrier remodelling and disease-conditioned neutrophil states in organ fibrosis. Neutrophils can influence extracellular matrix degradation, matrix transport, local matrix organisation, barrier stability and stromal inflammatory responses; these activities may support early repair, but when persistent or dysregulated, they may contribute to a pro-fibrotic niche by amplifying inflammation, modifying matrix architecture and licensing fibroblast activation. The central conceptual advance is that neutrophils should be understood not as primary fibrogenic cells, but as transient, context-dependent structural regulators that can initiate, modify or amplify tissue remodelling according to timing, tissue niche and inflammatory persistence. Because much of the mechanistic evidence derives from mouse models, we also discuss human translation and the need to selectively restrain maladaptive neutrophil programmes without compromising host defence or early repair.

## Introduction

Neutrophils are among the most abundant leukocyte populations in human peripheral blood. They are traditionally defined as short-lived, terminally differentiated myeloid effector cells that are rapidly recruited during acute inflammation to eliminate pathogens and limit tissue damage through chemotaxis, adhesion, phagocytosis, degranulation, reactive oxygen species (ROS) production and neutrophil extracellular trap (NET) formation (1, 2). This classical framework remains central to host defence, but it does not fully explain the diverse roles of neutrophils in sterile injury, tissue repair and chronic inflammatory disease. Increasing evidence indicates that neutrophils are not a functionally uniform population of terminal effectors; instead, they display context-dependent plasticity shaped by developmental state, tissue localisation, timing and disease-specific microenvironmental cues. Depending on these conditions, neutrophils may amplify tissue injury, support inflammatory resolution, facilitate repair or contribute to pathological remodelling (3, 4).

After tissue injury, circulating neutrophils are rapidly recruited to damaged sites by chemokines, lipid mediators, damage-associated molecular patterns and other tissue-derived signals. These cues coordinate directional migration, endothelial adhesion, transendothelial migration and tissue infiltration through receptor signalling, cytoskeletal remodelling and integrin activation (5, 6).Once within injured tissues, neutrophils can further engage in intercellular coordination and local clustering, including neutrophil aggregation or swarming, which is reinforced by integrin signalling, leukotriene B4 and other chemotactic feed-forward loops (7–9). Thus, early neutrophil recruitment is not only a canonical host-defence response for infection control and damage clearance but may also shape the spatial immune architecture of injured tissues and influence the subsequent trajectory of inflammatory resolution, tissue repair and pathological remodelling.

A key emerging concept is that neutrophils can regulate tissue architecture. Beyond direct antimicrobial activity, neutrophils communicate with epithelial cells, endothelial cells, fibroblasts, macrophages and lymphocytes through proteases, cytokines, lipid mediators, NET-associated molecules and matrix-related proteins. Through these interactions, they can influence extracellular matrix (ECM) degradation, matrix transport, local matrix organization, vascular barrier stability and the transition from inflammation to repair (10–13). These functions are distinct from both the classical antimicrobial programme of neutrophils and the sustained matrix-producing programme of fibroblasts and other mesenchymal cells. Collectively, these context-dependent activities form the basis of what we refer to in this review as neutrophil-mediated structural immunity. Accordingly, neutrophils may be viewed as short-lived but highly responsive tissue-sculpting cells: they can support early structural reorganization and barrier restoration after injury, whereas persistent or dysregulated inflammatory cues may convert these activities into contributors to a pro-fibrotic stromal niche. Rather than replacing fibroblasts as the principal matrix-producing cells, neutrophils may promote pathological remodelling by modifying ECM architecture, amplifying inflammatory signals and licensing fibroblast activation.

**Figure 1:**
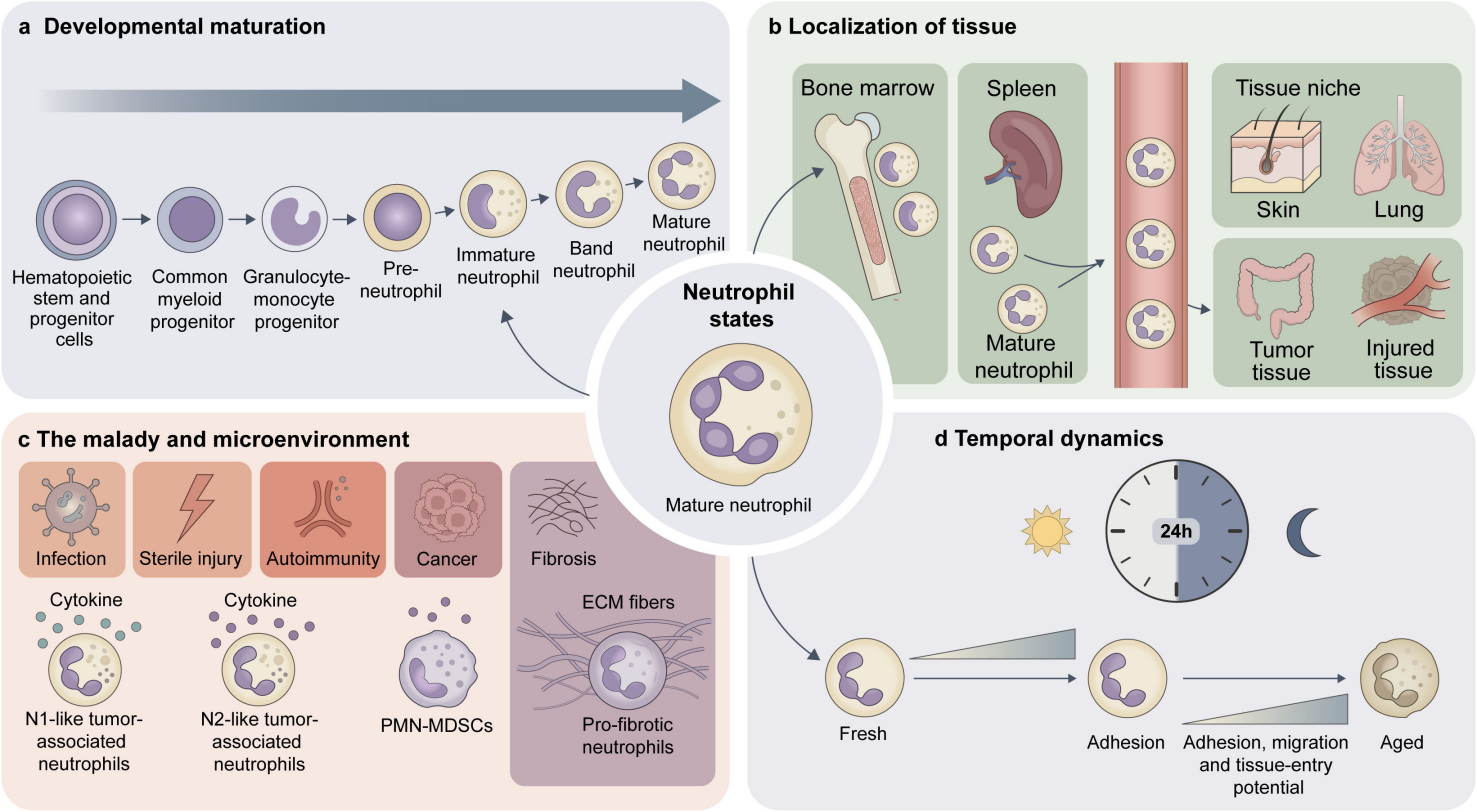
Multidimensional organization of neutrophil states. Neutrophil heterogeneity is a property of a continuum of different biological dimensions rather than fixed cellular subtypes. **(a)** Developmental maturation. Neutrophils develop from hematopoietic stem and progenitor cells through granulopoiesis that provides common myeloid progenitors, granulocyte-macrophage progenitors, pre-neutrophils, immature neutrophils, band neutrophils and segmented neutrophils. This maturation process is identified by progressing nuclear remodelling and gaining effector competence. **(b)** Localisation of tissue. Different anatomical compartments feature distinct neutrophil states. Bone marrow contains greater levels of immature and precursor populations, whilst the circulation contains mature neutrophils which can enter tissue niches, e.g., skin, lung, tumour tissue and injured tissue. **(c)** The malady and microenvironment. In going through different types of conditions, these different environments provide distinct inflammatory and stromal cues. Thus, this creates specialized neutrophil states which range from N1-like to N2-like. **(d)** Temporal dynamics. Circulating neutrophils gradually transition over time to different phenotypes and display different functional properties. These include transition from fresh neutrophils to aged neutrophils. Also, adopt a rhythmic pattern of adhesion, migration and tissue-entry potential. The inputs that are spatial, temporal, and disease-specific converge to define neutrophil states. The converged entity will determine the context-dependent roles of neutrophils. The roles may be in host defence, immune regulation, tissue repair, and pathological remodelling.

This duality raises a central question: how do neutrophils shift from reparative regulators of tissue architecture to drivers of maladaptive remodelling? In this review, we propose a broader “tissue-sculptor” conceptualization of neutrophils to better capture their non-canonical roles in tissue repair, barrier remodelling and fibrosis. This conceptualization should not be interpreted as a universal neutrophil programme operating uniformly across organs, but rather as a framework for examining how neutrophils acquire structural functions in defined tissue, temporal and disease contexts. Rather than providing a comprehensive survey of neutrophil biology, we focus on how canonical effector mechanisms can generate reparative or injurious structural outcomes, how neutrophils interact with the extracellular matrix (ECM) during wound repair and barrier maintenance, and how disease-conditioned neutrophil states contribute to organ fibrotic remodelling. By integrating these lines of evidence, this review aims to clarify how neutrophils connect inflammation, structural reconstruction and long-term tissue outcomes after injury. The multidimensional organization of neutrophil states is summarized in **Figure 1**.

## Neutrophil states relevant to tissue remodelling

The dimensions of neutrophil heterogeneity discussed below—including developmental position, tissue localisation, temporal regulation and microenvironmental conditioning—are relevant to tissue repair because they influence how neutrophils respond to injury and participate in matrix remodelling. Under steady-state conditions, neutrophils are generated predominantly through continuous granulopoiesis in the bone marrow; during infection, inflammation or haematopoietic stress, extramedullary myelopoiesis in organs such as the spleen can also contribute to neutrophil production. Neutrophil development begins with the stepwise differentiation of haematopoietic stem and progenitor cells into common myeloid progenitors and granulocyte–monocyte progenitors. At these early stages, myeloid progenitors retain the potential to generate multiple myeloid lineages, including monocytes and macrophages, neutrophils, eosinophils and basophils. Progressive lineage restriction then channels these cells into a neutrophil-committed developmental programme (14–16). In the classical morphological framework, neutrophil maturation proceeds through a continuum of myeloblasts, promyelocytes, myelocytes, metamyelocytes, band cells and segmented mature neutrophils. Promyelocytes and myelocytes constitute the main mitotic compartment of neutrophil development, with myelocytes generally regarded as the last morphologically defined stage that retains proliferative capacity. After the metamyelocyte stage, neutrophil-lineage cells lose mitotic activity and enter the post-mitotic compartment, marking the onset of terminal maturation (17). The following process of nuclear remodelling, granule composition alterations, changes in receptor expression for migration- association and acquisition of mature effector functions leads to the sequential formation of band cells and segmented mature neutrophils. In people, the post-mitotic maturation period from the last division at the metamyelocyte stage to the band cell transition into mature segmented neutrophils takes about 5–6 days whereas in rodents this process is much shorter, lasting about 2–3 days (18, 19).

Neutrophil development does not appear to follow a series of discrete morphological categories but rather a continuous development in maturation without a discernable branching point evident in the datasets. Merging neutrophils obtained from *in vivo* tissues and *in vitro* differentiation systems, researchers have reconstructed a “neutrotime” developmental trajectory in time order. The continuum of neutrophils occurs from proliferative neutrophil-committed pre-neutrophils, non-proliferative immature neutrophils, and finally mature neutrophils mainly.

Notably, neutrophils at distinct points along this developmental continuum are not seen evenly distributed in different tissue types. Rather, they show strong tissue-specific enrichment. In healthy mice, pre-neutrophils are found predominantly in the bone marrow, with only small numbers detectable in the spleen and very few present in the peripheral blood. Splenic neutrophils are largely positioned towards the mature end of the trajectory, although a substantial fraction remains less mature than circulating neutrophils. By contrast, neutrophils in the peripheral blood of healthy mice are almost exclusively located at the most mature end of this continuum, representing highly mature circulating neutrophil states. Nevertheless, the extent to which this mouse neutrophil maturation trajectory maps onto human neutrophil states remains to be fully defined (20).

During steady-state granulopoiesis, neutrophils undergo pronounced nuclear remodelling. In the classical morphological framework, neutrophil development is defined largely by nuclear morphology and granule characteristics, and is divided into successive stages that include promyelocytes, myelocytes, metamyelocytes, band neutrophils and segmented mature neutrophils. As cells progress from proliferative precursors into the post-mitotic maturation compartment, their nuclei evolve from relatively round or kidney-shaped structures into band-shaped forms and ultimately into segmented nuclei. In parallel, developing neutrophils progressively assemble their granule proteome, remodel surface receptor expression, acquire migratory competence and mature antimicrobial effector functions, thereby establishing a more complete host-defence programme. Functionally, immature neutrophils are not simply less mature versions of segmented neutrophils, but can display distinct effector and immunoregulatory properties (21, 22). However, whether these functional differences are directly determined by nuclear morphology remains unclear. A more plausible interpretation is that nuclear segmentation marks the developmental position of neutrophils, whereas functional divergence is shaped by the maturation of granule stores, surface receptor repertoires, transcriptional and metabolic states, and tissue-derived microenvironmental signals (23). Thus, nuclear morphology should be considered an important indicator of neutrophil maturation, rather than the sole determinant of neutrophil function.

temporal regulation involves not only immune effector cells, but also structural cells that spatially organize and coordinate immune responses. In both humans and mice, circulating granulocytes and monocytes show circadian fluctuations in abundance, and T cells and B cells also exhibit marked time-dependent variation (24). These observations indicate that the immune system does not operate in a constant state but is instead shaped by multidirectional circadian circuits involving the central clock, peripheral tissue clocks, structural elements and immune cells. Palomino-Segura and colleagues proposed that interactions among the central circadian clock, tissue structural components and immune cells form complex rhythmic networks that influence the efficiency, magnitude and timing of immune responses and other physiological processes (25).

At the molecular level, circadian variation in clock gene expression has been documented across multiple immune cell types, including monocytes, macrophages, neutrophils, dendritic cells and lymphocytes (26). Circulating neutrophils, in particular, undergo natural fluctuations in phenotype and function over the course of the day, including rhythmic changes in adhesive capacity, chemotactic responsiveness, inflammatory activity and tissue-homing propensity (27).These findings suggest that neutrophils are not maintained in a static mature state, but can undergo qualitative remodelling over time in response to changing physiological demands (28). Thus, circadian regulation may influence not only the abundance of neutrophils in the circulation, but also their ageing, tissue entry, local effector functions and contribution to tissue homeostasis.

One important consequence of circadian regulation in innate immunity is that inflammatory susceptibility, immune response intensity and the risk of tissue damage vary across the day. Temporal gating of immune responses may help balance host defence with tissue protection, thereby optimizing antimicrobial immunity while limiting unnecessary collateral injury. Conversely, when circadian rhythms are disrupted or immune clocks become misaligned with environmental cues, neutrophil responses may be amplified or mistimed, thereby aggravating inflammatory tissue damage. Circadian rhythmicity should therefore be considered an important temporal dimension of neutrophil heterogeneity, with implications not only for disease-associated inflammation but also for tissue homeostasis and barrier maintenance under steady-state conditions (29).

Neutrophil heterogeneity should not be viewed simply as the existence of fixed “subtypes”, but rather as a dynamic spectrum of cellular states shaped by developmental stage, tissue localisation, inflammatory cues and disease-specific microenvironments. This spectrum ranges from immature neutrophils that reside in the bone marrow and can be rapidly mobilized into the circulation, to neutrophil states with distinct transcriptional profiles and regulatory functions in infection, sterile injury, autoimmune disease and cancer. These different states can exert divergent effects on tissue homeostasis, inflammatory responses and disease progression (30, 31).

Tumour-associated neutrophils provide a prominent example of how disease-specific microenvironments generate functionally divergent neutrophil states. Early frameworks distinguished antitumour N1-like from protumour N2-like states, whereas subsequent studies identified polymorphonuclear myeloid-derived suppressor cells as immunosuppressive populations that overlap with some protumour neutrophil states (32) (33). However, these categories do not capture the full continuum of tumour-associated neutrophil phenotypes and should not be treated as interchangeable. In the context of this review, their relevance lies primarily in illustrating how local microenvironmental cues can condition neutrophil–stromal interactions and thereby potentially influence extracellular matrix remodelling and fibroblast activity.

Beyond cancer, circulating neutrophils themselves are not a uniform population. Diverse disease states and systemic stress conditions, including cancer, infection, sterile inflammation and haematopoietic reconstitution, can release neutrophils of different maturation stages and functional states into the peripheral blood (34) (30, 35). Under these conditions, immature or stress-induced neutrophils may differ in their capacity for reactive oxygen species production, neutrophil extracellular trap formation, cytokine release and immunoregulatory activity. These observations suggest that circulating neutrophils are not merely terminal effector cells passively released from the bone marrow but may undergo a form of environmental “education” during maturation and trafficking.

Thus, neutrophil heterogeneity can be understood as the integrated result of a cell’s starting position along the neutrophil maturation continuum, the nature of the microenvironmental signals it encounters and the temporal sequence of these exposures (20). Together, these variables generate a functionally diverse neutrophil pool in which distinct cellular states are primed to participate in pathogen clearance, inflammatory amplification, immune suppression, tissue repair or pathological remodelling in a context-dependent manner (36). This framework provides the basis for understanding why neutrophils can exert reparative, matrix-remodelling or pro-fibrotic effects in different tissue and disease settings.

## Canonical effector mechanisms as structural regulators

Neutrophils contribute to host defence not only by phagocytosing and killing pathogens, but also by communicating with macrophages, dendritic cells and adaptive immune cells through direct cell–cell contact and soluble mediators, thereby shaping the local immune microenvironment (37).Their classical effector functions are initiated by the recognition of pathogens and opsonizing host signals. Neutrophils express multiple classes of cell-surface and intracellular receptors that directly detect microbial-associated molecular patterns, as well as Fc receptors and complement receptors that recognize pathogens opsonized by immunoglobulin G or complement components. Together, these receptor systems activate intracellular signalling pathways that enable or enhance chemotaxis, phagocytosis, oxidative burst, degranulation and cytokine release.

Importantly, the magnitude, nature and duration of neutrophil responses are not dictated by a single receptor pathway but are shaped by the receptor repertoire engaged within a given spatial and temporal context. This input from a variety of receptors is what would help define the functional “set point” of neutrophils, that is, whether they remain quiescent, become primed or undergo full activation (38). Neutrophils primarily detect microbes and danger-associated patterns through the Toll-like receptor (TLR) family of non-phagocytic pattern-recognition receptors. Neutrophils express TLR1, TLR2, TLR4, TLR5, TLR6, TLR8 and TLR10 at the RNA level; TLR9 expression can also be induced after stimulation with granulocyte–macrophage colony-stimulating factor. They can recognize a range of potential ligands like lipids, carbohydrates, peptides, DNA and single-stranded or double-stranded RNA. According to TLR activation does not always trigger the full effector programme but often primes neutrophils to engage in stronger responses when subsequently stimulated. This enhances their phagocytic and antimicrobial capacity, cytokine release and delays apoptosis (39). Consequently, signal integration through receptor-mediated processes forms an important connection between pathogen recognition and effector amplification as well as immunoregulatory functions of neutrophils.

After infection or injury to tissue, the local inflammatory mediators activate the endothelial cells of the blood vessels and form a luminal recruitment platform of selectins, adhesion molecules and chemokines. Before migrating toward an inflammatory stimulus, circulating neutrophils first roll along the endothelial surface. This process can be classified as primary and secondary capture, depending on the partners and adhesion molecules involved. The initial contact between neutrophils and activated endothelial cells is referred to as primary capture and is quite distinct from secondary capture which is mainly mediated by the action of L-selectin allowing neutrophils to interact with already rolling leukocytes or leukocyte-derived structures (40). Following this interaction, neutrophils will start sustained but progressively slower interactions with the endothelial surface which is termed rolling that allows for a time window helpful for sensing endothelial chemokines and inflammatory signals (41).

Under conditions of high haemodynamic shear stress, rolling leukocytes can form membrane tethers and slings that increase the adhesive contact area with the endothelial surface and stabilize rolling behaviour (42). In severe inflammatory settings, such as sepsis, persistent high shear stress and marked endothelial activation can promote the detachment of these membrane structures from neutrophils, generating elongated neutrophil-derived structures enriched in S100A8/A9 (43).Depending on local calcium concentrations, these structures can release S100A8/A9 into the surrounding microenvironment, thereby reinforcing an inflammatory endothelial surface and potentially amplifying subsequent leukocyte recruitment and local inflammation (44).

During rolling, chemokines presented on the endothelial surface induce conformational activation of neutrophil integrins, converting them from low-affinity to high-affinity states. High-affinity lymphocyte function-associated antigen(LFA)-1 interacts with its endothelial ligand intercellular adhesion molecule 1 (ICAM-1), causing neutrophils to arrest from slow rolling and adhere firmly to the endothelial surface (45, 46).In parallel, engagement of the β2 integrin macrophage-1 antigen with ICAM-1 further stabilizes adhesion and contributes to subsequent intraluminal crawling, transendothelial migration and tissue infiltration (47).In addition to the β2 integrins LFA-1 and Mac-1, β1 integrins can also regulate neutrophil adhesion; for example, very late antigen 6 (VLA-6) binds laminin, whereas VLA-4 interacts with vascular cell adhesion molecule (VCAM) - 1 (48).Thus, vascular recruitment of neutrophils is not a single adhesive event, but a sequential adhesion cascade driven by selectin-mediated capture and rolling, chemokine-induced integrin activation, and coordinated engagement of multiple integrin–ligand axes.

After firm adhesion to the endothelial surface, neutrophils can move slowly along the luminal side of the vessel wall, a process known as intraluminal crawling. This behaviour enables neutrophils to scan the endothelial surface for permissive sites of transendothelial migration, and their movement can occur either with or against the direction of blood flow. Reverse migration is conceptually distinct from crawling and refers to the process by which neutrophils that have completed, or partially completed, transendothelial migration return from the abluminal or subendothelial compartment to the vascular lumen and re-enter the circulation (49). Notably, even neutrophils that have executed effector functions such as phagocytosis can re-enter the bloodstream, indicating that local effector activation does not necessarily preclude subsequent reverse migration.

A related phenomenon is “hesitant migration”, in which neutrophils initiate extravasation but fail to complete transendothelial migration, instead disengaging from the endothelium or returning to the luminal compartment (50). Neutrophils that undergo hesitant migration or reverse migration may remain activated or primed and could therefore transmit inflammatory signals to vascular beds distant from the primary site of injury, contributing to secondary inflammatory responses.

The patterns of neutrophil recruitment and migration are also organ-specific and vascular bed-specific. In many microvascular settings, the β2 integrin Mac-1 is a key regulator of neutrophil intraluminal crawling on ICAM-1 (51). However, on primary mouse brain microvascular endothelial cells, neutrophil crawling is not governed by a single integrin axis, but is jointly regulated by interactions of LFA-1 and Mac-1 with their ligands ICAM-1 and ICAM-2 (52, 53).These findings indicate that pre-transendothelial neutrophil motility is not a fixed programme, but is shaped by local endothelial phenotypes, haemodynamic conditions and the specific combination of integrin–ligand interactions.

The classical antimicrobial programme of neutrophils is built around three tightly coupled processes: phagocytosis, oxidative burst and the release of granule contents. After the binding of pathogens by Fcγ receptors, complement receptors, C-type lectin receptors and other pattern-recognition receptors, neutrophils quickly engulf microbial particles. Also, they internalize them into pathogen-containing phagosomes (54). Neutrophils as professional phagocytes not only kill invading microorganisms, but also clear away cellular debris and degradative material (55). This process is very active. For instance, the uptake of IgG-opsonized particles can take place within several tens of seconds. The phagosome then fuses with preformed intracellular granules and matures. The first of these, the presence of granule-derived hydrolases, antimicrobial peptides, proteases and components of the nicotinamide adenine dinucleotide phosphate (NADPH) oxidase complex contrives a very toxic environment in the phagosome, with the generation of reactive oxygen species that facilitate the release of antimicrobial mediators for degradation and clearance of the pathogen (56).

Nevertheless, the process of phagocytosis and maturation of the phagosome is not always limited to only a safe intracellular killing compartment. When the granules are incorporated into the phagosome and fuse before complete sealing, the contents of the granules and other oxidative products may leak into the extracellular space damaging the neighbouring cells and tissues (57). Neutrophils in some environments may get “frustrated phagocytosis”. When immune complex or complement deposition occurs on large tissue surfaces, neutrophil phagocytic receptors can be engaged without complete internalization of the target. Under these conditions, granule proteins, proteases and oxidative products are released extracellularly, causing widespread tissue injury. Thrombohaemorrhagic vasculitis provides a representative example of this mechanism: inflammatory mediators such as tumour necrosis factor promote extensive complement component 3 (C3) deposition on the vascular endothelium, which activates neutrophils through complement receptor 3 (CR3)/Mac-1, induces massive granule release and ultimately results in vascular destruction and haemorrhage (58). Thus, spatial misdirection of neutrophil killing programmes is an important source of tissue injury in infectious and sterile inflammatory diseases.

Reactive oxygen species are central effector molecules in neutrophil antimicrobial responses, but their functions extend beyond direct microbial killing. High levels of reactive oxygen species (ROS) contribute to pathogen destruction within phagosomes, whereas low-level or spatially restricted ROS can function as signalling molecules that regulate neutrophil migration and polarity (1, 59, 60). For example, low levels of ROS may inactivate the phosphatase PTEN, thereby promoting the accumulation of phosphatidylinositol-3, 4, 5-trisphosphate at the leading edge of migrating cells and enhancing chemotactic responses (61). On the contrary, inhibition of ROS generation may preserve or inhibit directional neutrophil migration, indicating a dose, subcellular localisation and stimulus context dependent effects of ROS on motility (62).

Neutrophils also have effector programmes for degranulation. When neutrophils release elastase, matrix metalloproteinases, cathepsins and other granule proteins, they degrade extracellular matrix, promote leukocyte infiltration, and reshape local tissue architecture (63).This process is double-edged. Spatially restricted breakdown of the matrix can promote the entry of immune cells into injured tissue and assist in the clearance of pathogens and dead tissue. By contrast, unfettered release of granule content can damage barrier structures, worsen tissue damage and promote inflammation detrimental to health. Degranulation is not intrinsically destructive; its effects depend on the timing, magnitude, spatial localisation and tissue context of granule release. The neutrophils coordinate the myocardial healing in different models of myocardial infarction by facilitating the macrophage polarization towards repair-associated phenotypes (64). Myocardial infarction influences cardiac neutrophils which experience selective degranulation during its temporal course. The proteomic state of neutrophils and the ECM environment dictate selective degranulation. Neutrophil degranulation signatures are closely related to matrix metalloproteinases, cathepsins and extracellular matrix-associated proteins, fibronectin, fibrinogen, galectin-3, thrombospondin-2 and vitronectin. Among these, fibronectin acts as an important regulator of degranulation and amplifies neutrophil matrix metalloproteinase 9 release in the presence of inflammatory stimulation (65).

Taken together, phagocytosis, ROS generation and degranulation constitute the core modules of the classical neutrophil antimicrobial programme. When these effector mechanisms are tightly constrained in space and time, they promote pathogen clearance, removal of necrotic material and initiation of repair. When they become extracellularly misdirected, persistently activated or improperly localized, the same killing machinery can become a driver of tissue injury, barrier disruption and pathological remodelling.

During antimicrobial defence, neutrophils can release neutrophil extracellular traps (NETs), which capture pathogens, limit their dissemination and enhance local microbial killing. NETs are web-like structures composed of decondensed chromatin decorated with granular, cytoplasmic and nuclear proteins, and can entrap a broad range of microorganisms, including bacteria, viruses and fungi (66). In specific immune contexts, nucleoprotein immune complexes can also induce NET formation through FcγRIIIb-mediated signalling in neutrophils primed by type I interferons (67).Thus, NETs represent an important component of the classical antimicrobial neutrophil response, while also functioning as effector structures that connect innate immune sensing, local inflammatory amplification and tissue injury.

However, when immune responses become dysregulated, excessive or mislocalized NET formation can amplify inflammation and damage host tissues, thereby contributing to the development and progression of diverse infectious and sterile inflammatory diseases. In pathological sterile inflammation, NET formation can be induced by multiple host-derived signals, including the receptor for advanced glycation end-products (RAGE), the P-selectin–P-selectin glycoprotein ligand 1 (PSGL1) axis, TLRs, low-affinity IgG Fc receptors (FcγRs) and sialic acid-binding immunoglobulin-like lectins (Siglecs). These pathways indicate that NET formation is not restricted to direct antimicrobial defence but can also be driven by damage-associated molecules, vascular inflammation, immune complexes and cell–cell interactions.

NETs can associate inflammation with thrombosis because of procoagulant mechanism. They induce the release of tissue factor from activated platelets and monocytes and provide a physical scaffold for the adhesion of platelets, deposition of fibrin and aggregation of thrombotic molecules which enhance local procoagulant activity. Besides, accumulated NETs may block tubular structures like the bile ducts and pancreatic duct, leading to organ dysfunction and intensifying inflammation further in the locality (68). NETs should not be seen solely as antimicrobial structures, but rather as versatile effector platforms with extraordinary potential to contain pathogens, enhance inflammation, induce coagulation and damage tissues.

**Figure 2:**
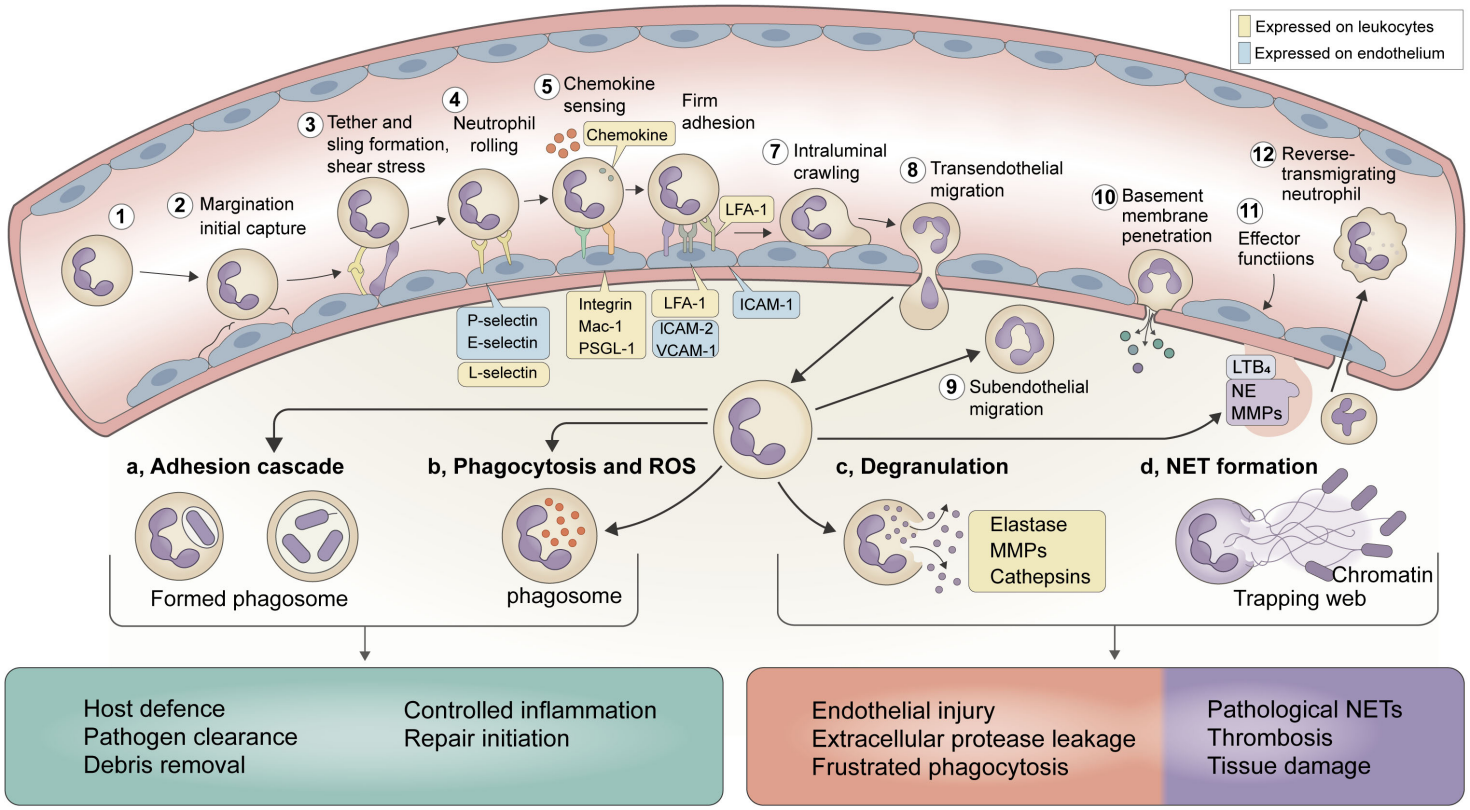
Classical neutrophil effector programmes balance host defence and tissue injury. Neutrophil recruitment undergoes several steps which include margination as well as capture along with selectin-mediated rolling, firm adhesion as well intraluminal crawling. After this, transendothelial migration is followed by subendothelial migration along with basement membrane penetration. In certain areas, activated neutrophils can transit in the reverse direction and return to the vascular lumen. Once they enter the tissue, neutrophils carry out classical effector programmes like phagocytosis, NET formation, etc. These mechanisms support host defence, debris clearance, inflammation and injury repair at a tightly-controlled level. On the contrary, these events become excessive and mislocalized due to endothelial injury, protease leakage, and other events.

Crucially, only certain populations of neutrophils are predisposed to NET formation. Neutrophils in various maturation stages, from diverse tissue compartments or in different disease-associated states may exhibit differences in their capacity to form NETs and the NETs generated by them may differ in composition and architecture. The quantity and quality of NET formation can affect the intensity, duration and nature of the inflammatory response (69). This supports the idea that classical neutrophil effector functions are not fixed properties but rather shaped by the developmental status microenvironmental cues and disease context. The major canonical effector programmes and their structural consequences are summarized in **Figure 2**.

## Context-dependent structural immunity mediated by neutrophils

For a long time, neutrophils were regarded as short-lived effector cells of acute inflammation, equipped to migrate, ingest, degranulate and produce reactive oxygen species and neutrophil extracellular traps. Over the past decade, this perspective has broadened. Studies in defined experimental systems have shown that neutrophils can also participate in tissue reconstruction by regulating pre-existing ECM, generating local matrix and barrier structures, and shaping fibrotic responses during chronic inflammation. In this review, we use the term “structural immunity” to describe context-dependent neutrophil activities that influence tissue architecture through the transport, organization or remodelling of extracellular matrix, reinforcement of physical barriers and modulation of stromal-cell responses.

**Figure 3:**
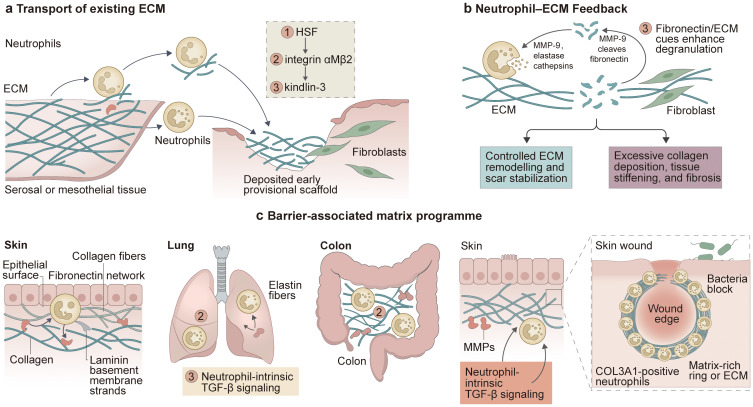
Neutrophil-mediated structural immunity and extracellular matrix remodelling. Neutrophils are involved in the reconstruction of tissues through structural programmes beyond classical antimicrobial defence. **(a)** Transport of existing ECM. Neutrophils can attach existing ECM fragments and move them to a damaged surface after serosal or mesothelial injury. This occurs via an HSF-integrin aMβ2-kindlin-3 axis. It can create an early provisional scaffold that helps fibroblast recruitment, matrix remodelling and stabilization of the wound. **(b)** Neutrophil–ECM feedback. ECM remodelling takes place by proteases released from neutrophils while ECM-derived cues like fibronectin fragment can promote neutrophil de-granulation. Under control, this feedback leads to matrix remodelling and stabilisation of the scar; when uncontrolled, it may lead to collagen accumulation, stiffening of the tissues and fibrosis. **(c)** Barrier-associated matrix programme. In a tissue with a barrier like the skin, lung and colon, neutrophils can express or organize matrix-associated components including collagens, fibronectin, laminins, elastin and matrix metalloproteinases. In skin wounds, neutrophils that are positive for COL3A1 build up at the wound edge to create a ring rich in matrix constituents that limits bacterial ingress and bolsters barrier integrity. This process is driven by TGF-β signalling intrinsic to neutrophils.

This concept does not imply a universal neutrophil programme or identify neutrophils as the principal matrix-producing cells in tissue repair or fibrosis. However, neutrophil-mediated structural immunity should be interpreted as a context-dependent conceptual framework rather than as a universal programme operating uniformly across organs. The strongest current evidence comes from murine visceral and serosal injury models, barrier-tissue models, myocardial infarction and renal fibrosis. These studies indicate that neutrophils can acquire matrix-transporting, matrix-producing or pro-fibrotic functions under defined tissue and disease conditions. Whether these activities represent conserved neutrophil programmes across diverse organs, injury types and species remains incompletely established. Mechanisms directly demonstrated in specific models should therefore be distinguished from broader interpretations that remain inferential. Within this framework, neutrophils may function as carriers of pre-existing ECM, contributors to matrix production or organization, matrix-remodelling inflammatory cells, or amplifiers of fibroblast activation. In other settings, neutrophil enrichment or disease-associated signatures may primarily reflect tissue inflammation rather than a direct structural function. These roles are related but not interchangeable, because they involve distinct mechanisms and are supported by different levels of causal evidence. Matrix association must also be distinguished from *de novo* matrix production. Matrix-related transcripts or proteins detected in neutrophils may reflect endogenous synthesis, transport of pre-existing ECM, surface adhesion, phagocytosed extracellular material or contamination from surrounding stromal cells. Evidence for neutrophil-derived matrix should therefore rely on orthogonal approaches that distinguish transcription and protein synthesis from secretion, extracellular deposition and matrix assembly. In the absence of such evidence, terms such as “matrix-associated programme” or “matrix-organizing activity” are more appropriate than claims of direct matrix production. The main forms of neutrophil–ECM interaction and matrix-related structural immunity are summarized in **Figure 3**. The evidence supporting these structural roles across tissue and disease contexts is summarized in **Table 1**.

**Table 1:** Neutrophil structural functions across tissues and disease contexts.

Structural role / context	Species / model	Evidence type and level	Main finding	Human validation	Confidence	Key reference
Carrier of pre-existing ECM Early visceral/serosal repair	Mouse peritoneal, hepatic and caecal mesothelial injury	In vivo tracking and HSF–integrin αMβ2–kindlin-3 perturbation; mechanistic	Neutrophils relocate pre-existing ECM to injured surfaces and form an early provisional scaffold.	None	High within these models; uncertain generalizability	Fischer et al. (13)
Matrix-producing or matrix-organizing cell Barrier maintenance and wound protection	Primarily mouse skin; related states reported in lung and colon	Single-cell/spatial analyses and neutrophil-specific TGF-β perturbation; mechanistic in skin	Neutrophils contribute to skin ECM organization, mechanical integrity and a wound-edge matrix barrier.	Limited	High in mouse skin; uncertain in other tissues and humans	Vicanolo et al. (12)
Matrix-remodelling inflammatory cell Myocardial infarction	Mouse myocardial infarction models	Time-course proteomics and depletion studies; mixed mechanistic/associative	Early states support ECM degradation and debris clearance; later states contribute to matrix reorganization and scar stabilization.	Indirect	Moderate	Horckmans et al.; Ma et al.; Daseke et al. (64, 65, 85)
Amplifier of fibroblast activation Periodontitis-associated post-MI fibrosis	Mouse periodontitis plus myocardial infarction	Single-cell analysis, subset depletion and fibroblast assays; mechanistic in mice	Siglec-F^+^ neutrophils promote fibroblast activation and collagen-associated remodelling.	Human gingival observations are associative	Moderate-to-high in mice; low for human correspondence	Wang et al. (89)
Amplifier of fibroblast activation Renal fibrosis	Mouse UUO, adriamycin nephropathy and renal ischaemia–reperfusion injury	Subset depletion, adoptive transfer and in vitro induction; mechanistic in mice	Siglec-F^+^ neutrophils promote fibroblast activation and a collagen-associated programme, including increased COL1A1 expression.	Human Siglec-expressing observations are associative	High for mouse disease promotion; low for human equivalence	Ryu et al. (90)

Confidence refers to the stated model and does not imply cross-tissue or cross-species generalizability. ECM, extracellular matrix; MI, myocardial infarction; UUO, unilateral ureteral obstruction.

## Neutrophil–matrix interactions in early repair

Early tissue repair requires rapid ECM accumulation before the injured site is replaced by more stable anatomical structures that preserve organ integrity and organismal survival. Failure of this process can result in chronic non-healing wounds, scarring or progressive fibrosis (70–72), highlighting the importance of matrix deposition for the restoration and maintenance of organ function. Traditionally, wound-associated ECM was thought to be produced mainly by fibroblasts that migrate into the wound bed and locally synthesize and deposit matrix. This model remains valid, but it does not fully explain the rapid matrix accumulation observed during the earliest phases of visceral injury. Fibroblasts are central matrix-producing cells and interact extensively with immune and stromal compartments through reciprocal signalling (73–77); however, recent studies indicate that neutrophils also contribute to early matrix organization by regulating fibroblast-dependent deposition and, in some contexts, by degranulation-associated matrix production (65, 78).

A key study by Fischer and colleagues showed that neutrophils can transfer pre-existing ECM to injured sites. In mouse models of peritoneal, hepatic and caecal mesothelial injury, matrix appearing in the wound shortly after damage was not derived entirely from newly synthesized ECM but was partly mobilized from distant organ surfaces. Over time, this transferred matrix was remodelled by newly synthesized matrix, and ECM from distinct anatomical sites became crosslinked within the repairing wound, contributing to mature, stable and interconnected fibrous adhesions. Importantly, this early matrix transfer depended primarily on neutrophils rather than on early fibroblast migration (13). Neutrophil recruitment after injury is coordinated by leukotrienes, lipoxin A4, chemokine–chemokine receptor axes including C-X-C chemokine receptor 2 (CXCR2) and CXCR4, and nitric oxide-dependent pathways (7, 60). Mechanistically, matrix transfer was reported to depend on a heat shock factor (HSF)–integrin αMβ2–kindlin-3 axis in neutrophils. HSF-dependent signalling appears to promote αMβ2 integrin activation in wound-associated neutrophils, whereas kindlin-3 functions as a haematopoietic integrin adaptor required for firm cell–matrix adhesion (79–81). This pathway enables neutrophils to adhere to and transport pre-existing extracellular matrix during early repair. After entering the wound, these neutrophils acquire injury-associated states and ultimately undergo apoptosis. Blocking this pathway prevented matrix transfer, scar formation and peritoneal adhesions in mice, suggesting potential therapeutic relevance (13). Thus, neutrophil-transported pre-existing ECM functions as an early provisional scaffold that enables subsequent repair. Neutrophils, apart from serving as inflammatory effector cells, may act as matrix carriers in the early stages of wound repair. In this paradigm, neutrophils do not primarily create the initial wound scaffold through *de novo* collagen synthesis but rather relocate existing ECM to the wound to provide a precondition for fibroblast activation, matrix remodelling and scar maturation. In this setting, neutrophils should therefore be regarded primarily as carriers of pre-existing ECM rather than as *de novo* matrix-producing cells. However, this mechanism has not yet been established as a general principle of wound healing. Its relevance to cutaneous repair, chronic non-healing wounds and organ fibrosis outside visceral or serosal contexts requires direct validation.

## Neutrophils in barrier repair and matrix organization

Beyond transporting pre-existing ECM, neutrophils may contribute to matrix production or organization in selected barrier-tissue models. The strongest current evidence comes from mouse skin, where neutrophils express a broad matrix-associated programme and contribute functionally to local ECM architecture and wound-edge barrier formation (12). Physical barriers provide mechanical protection against microbial invasion and environmental injury (82), whereas innate immune cells are classically thought to act after barrier breach by deploying cytotoxic antimicrobial mechanisms (83). Recent work has identified a population of neutrophils in barrier tissues, including lung, colon and skin, that expresses a broad ECM programme, encompassing collagens, fibronectin, laminins, elastin, matrix metalloproteinases and enzymes involved in fibre assembly and maturation. In steady-state skin, these neutrophils contribute to ECM composition and architecture. Their matrix programme is driven by neutrophil-intrinsic TGF-β signalling, enhances tissue mechanical strength and supports barrier function. This programme is also temporally regulated: many skin ECM genes exhibit circadian oscillation that coincides with the timing of neutrophil tissue entry. These observations suggest that neutrophils contribute dynamically to ECM homeostasis, although the relative contribution of direct versus indirect mechanisms requires further investigation, and circadian fluctuations in skin stiffness are attenuated, indicating that neutrophils dynamically regulate ECM homeostasis. After injury, a subset of COL3A1-expressing neutrophils forms a matrix-rich ring around the wound, functioning as an ECM “wall” that limits the entry of exogenous molecules and bacteria. This structure depends on neutrophil-intrinsic TGF-β signalling; blockade of neutrophil TGF-β receptor signalling impairs matrix-ring formation and increases bacterial invasion (12). The findings elaborate the functioning of neutrophils. Historically, barrier architecture has been ascribed to epithelial and stromal cells; however, matrix-producing neutrophils suggest that innate immune cells can reinforce barrier architecture directly. In selected mouse barrier-tissue models, neutrophils may therefore contribute not only to immune regulation but also to the organization of physical barrier structures. These findings support a direct structural role for neutrophils in selected barrier tissues, but the relative contribution of neutrophil-derived matrix compared with epithelial and stromal sources, and the relevance of this programme to non-barrier organs, remain to be determined.

## Disease-conditioned neutrophil states in fibrotic remodelling

The effects of neutrophils on ECM are not restricted to the initiation of repair. In normal condition neutrophils help in maintain barrier architecture and ECM remodelling (12). Nonetheless, during ongoing inflammation, those exact structural processes may become excessive and promote organ fibrosis. Following myocardial infarction (MI), neutrophils undergo a sequential functional transition. Early neutrophils are mainly pro-inflammatory, and degranulating and matrix degrading while later neutrophils are linked to resolution and ECM remodelling and scar stabilization. MI starts with cardiomyocyte necrosis, leading to inflammation and leukocyte infiltration, leading to scar formation. Neutrophils are the first responders that trigger post-MI inflammation (84) but also participate in inflammatory resolution and restraint (85). While neutrophil accumulation leads to tissue injury and detrimental left ventricular dilatation, neutrophil depletion would significantly worsen inflammation, ventricular dilatation and cardiac function (64, 86, 87). Thus, in the post-MI setting, neutrophils exert stage-dependent effects on cardiac repair and fibrotic remodelling.

The proteomic profiling of cardiac neutrophils during the first week after MI showed that the polarization state of neutrophils formed a continuum. On the first day following MI, the inflammatory response involving matrix metalloproteinase 8 (MMP-8) and MMP-9 is induced. These help with clearance of the necrotic tissue, ECM degradation and propagation of inflammatory signals. On day three, neutrophils exhibit increased levels of cathepsin activity signalling and apoptotic signalling, as well as generation of E.C.B. day 5, ECM reorganization is enhanced, neutrophil recruitment declines and inflammation resolves (65). By day 7, neutrophil numbers are low, but remaining neutrophils can still shape the emerging ECM microenvironment through matrix remodelling (85). These observations indicate that neutrophils cannot be classified simply as ECM-damaging or ECM-depositing cells; instead, they execute stage-specific matrix functions during repair. In this context, neutrophils are best classified as matrix-remodelling inflammatory cells whose proteolytic and degranulation programmes alter ECM composition in a time-dependent manner. Importantly, ECM also feeds back on neutrophil behaviour. Fibronectin selectively promotes neutrophil degranulation and, in the presence of pro-inflammatory stimuli, enhances MMP-9 release; MMP-9 in turn cleaves fibronectin into 120-kDa fragments, thereby altering local ECM composition (65). This creates a local neutrophil–ECM feedback loop in which neutrophils remodel ECM, and ECM reciprocally shapes neutrophil function. Such a loop may facilitate necrotic tissue clearance, scaffold renewal and scar stabilization, but if prolonged or amplified, it may provide a structural basis for chronic fibrosis (88).

**Figure 4:**
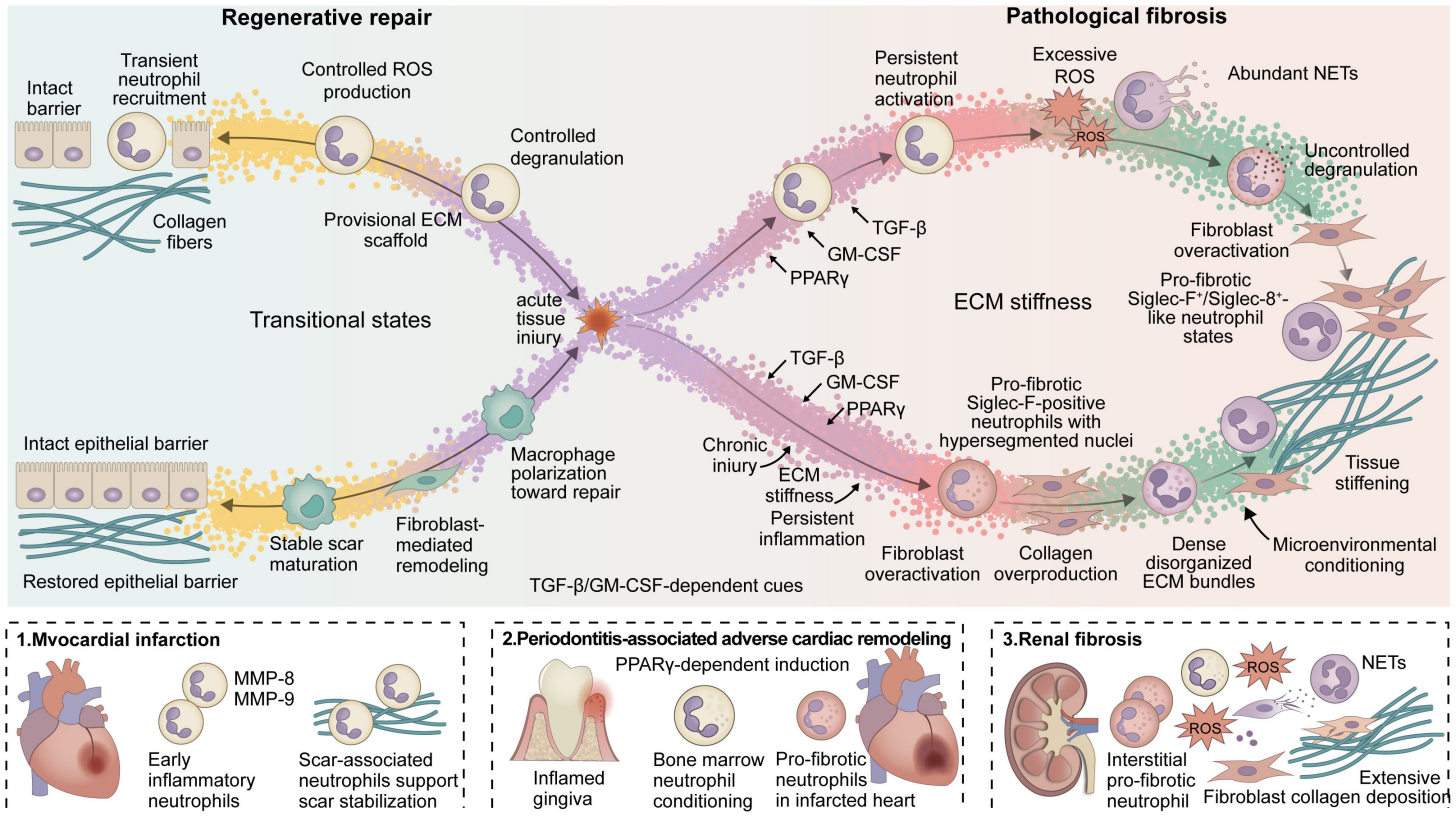
Context-dependent neutrophil structural programmes in regenerative repair and pathological fibrosis. The duration of inflammation, the environment of connective tissue and cues specific to disease lead to generation of different tissue outcomes by the neutrophil structural programmes. When injury is acute and self-limiting, recruitment of neutrophils, with controlled production of ROS, regulated degranulation, formation of provisional ECM scaffold, repair-associated macrophage polarisation, fibroblast-mediated matrix remodelling, barrier restoration and formation of stable and mature scar results. In these circumstances, neutrophils promote both inflammatory resolution and organized repair. In contrast, persistent inflammation alters neutrophil activity towards maladaptive remodelling. Ongoing activation, excessive production of reactive oxygen species, extensive formation of neutrophil extracellular traps, and unregulated release of cytotoxic substances can enhance activation of fibroblasts, excess collagen production, disorganization of extracellular matrix, stiffening of matrix, and fibrosis. Pro-fibrotic neutrophil states induced by disease may favourably strengthen the process under the influence of TGF-β, GM-CSF, PPARγ signalling. Contexts include myocardial infarction, where neutrophils mediate stage-specific matrix remodelling; adverse cardiac remodelling post-periodontitis, where chronic inflammation of the oral cavity may precondition neutrophil responses prior to cardiac injury; and renal fibrosis, where pro-fibrotic neutrophils, ROS, NETs and fibroblast activation drive progressive collagen accumulation.

Fibrosis should therefore not be regarded as a process entirely separate from neutrophil structural programmes. but neither should neutrophils be repositioned as primary fibrogenic cells. Fibrosis is a multicellular stromal process involving fibroblast activation, myofibroblast differentiation, matrix crosslinking, tissue stiffening, mechanotransduction, vascular injury, epithelial or endothelial dysfunction, macrophage-derived cues and failed inflammatory resolution. Within this broader network, neutrophils may contribute as early structural initiators, matrix modifiers, inflammatory amplifiers and fibroblast-licensing cells. During acute injury, neutrophil-mediated matrix mobilization, protease release, provisional scaffold formation and barrier stabilization may support tissue integrity and repair (12, 13). When inflammation persists, however, sustained ROS production, excessive NET formation, uncontrolled degranulation and disease-conditioned pro-fibrotic neutrophil states may reshape the extracellular matrix, amplify stromal inflammation and create conditions that favour fibroblast activation, myofibroblast differentiation, collagen deposition and matrix stiffening. Thus, neutrophils are best viewed as context-dependent regulators of the fibrotic niche rather than as the dominant matrix-producing cells in fibrosis. The context-dependent divergence between regenerative repair and pathological fibrosis is summarized in **Figure 4**.

This concept is supported by studies of Siglec-F^+^ neutrophils. Although Siglec-F has historically been used as a marker of mouse eosinophils, recent studies have identified Siglec-F^+^ neutrophil subsets in chronic inflammatory and fibrotic settings. These cells are often characterized by heightened activation, prolonged survival, increased ROS and NET formation, release of pro-fibrotic mediators and collagen-associated programmes. Their significance is not that all neutrophils produce collagen, but that neutrophils can acquire a pro-fibrotic structural state in specific tissue microenvironments (89, 90).

Periodontitis is a chronic oral inflammatory disease that is epidemiologically associated with adverse cardiovascular outcomes (91). Gingival sequencing studies show marked neutrophil enrichment in periodontitis and indicate that a matrix–neutrophil axis shapes gingival immunity (92). Blood neutrophils from patients with periodontitis often display hyperactivation and resistance to apoptosis, features that can persist even after extraction of periodontitis-affected teeth (93). Such primed neutrophils may predispose the host to exaggerated inflammatory responses after a second insult, such as MI (94). Together, these observations suggest that neutrophils, or specific neutrophil subsets, may contribute to cross-disease interactions between periodontitis and cardiovascular injury.

Wang and colleagues reported that chronic periodontitis aggravates cardiac fibrosis and functional deterioration after MI. In ligature-induced mouse periodontitis, two major neutrophil states were identified: Retnlg^+^ neutrophils, defined by expression of resistin-like gamma, and Siglec-F^+^ neutrophils, with the latter accounting for more than half of all neutrophils. In human gingival tissue, patients with periodontal disease showed a higher proportion of myeloid cells, and the human Siglec5 and Siglec14 genes, functional counterparts within the Siglec family, were highly expressed in myeloid cells. Mechanistically, periodontitis biased bone marrow neutrophil differentiation towards a scar-associated Siglec-F^+^ state, which accumulated in infarcted hearts, promoted fibrosis and impaired cardiac repair. This subset could be induced by granulocyte-macrophage colony-stimulating factor (GM-CSF) and/or TGF-β through a PPARγ-dependent mechanism and expanded within the infarcted heart. In the mouse periodontitis–MI model, Siglec-F^+^ neutrophils promoted cardiac fibrosis by activating fibroblasts and were reported to participate in collagen-associated remodelling. Local depletion of Siglec-F^+^ neutrophils reduced fibrosis and improved cardiac function; however, depletion also increased the risk of cardiac rupture after MI, probably owing to insufficient early collagen deposition and defective tissue repair. These findings identify Siglec-F^+^ neutrophils as key contributors to excessive post-MI fibrosis and cardiac dysfunction (89).

This apparent paradox is illustrated by two seemingly conflicting observations: broad neutrophil depletion after myocardial infarction can increase the risk of cardiac rupture, whereas selective depletion of Siglec-F^+^ neutrophils in experimental models has been reported to reduce fibrosis and attenuate adverse cardiac remodelling. These findings are not necessarily contradictory but instead highlight the time-dependent and subset-specific nature of neutrophil structural programmes. In the early phase after myocardial infarction, neutrophils may support necrotic tissue clearance, provisional matrix formation and mechanical stabilization of the injured myocardium; broad depletion at this stage may therefore impair repair and increase the risk of rupture. By contrast, persistent or disease-conditioned expansion of Siglec-F^+^ neutrophil states in fibrotic microenvironments may amplify collagen deposition, fibroblast activation and adverse remodelling. Thus, neutrophil structural activity is not intrinsically beneficial or harmful, but depends on timing, magnitude, subset identity and tissue context. Therapeutic strategies should therefore aim to restrain maladaptive neutrophil states without disrupting early reparative neutrophil functions.

The importance of this work lies in linking chronic oral inflammation, biased bone marrow myelopoiesis and adverse cardiac remodelling after MI. Periodontitis may not worsen MI outcomes solely by increasing systemic inflammatory markers; it may also pre-programme neutrophils to adopt a pro-fibrotic structural state after a second injury. Thus, cardiac fibrosis is not an incidental phenomenon outside neutrophil-mediated repair but may arise when reparative neutrophil programmes are biased by chronic inflammatory conditioning.

Renal fibrosis is a common pathological endpoint of chronic kidney disease and is characterized by excessive accumulation of ECM proteins, particularly type I collagen (95). Infiltrating immune cells promote renal fibrosis by producing pro-fibrotic cytokines, including TGF-β1, IL-1, IL-4 and IL-6, as well as growth factors such as platelet-derived growth factor and fibroblast growth factor 2. These immune responses may initially support renal repair, but persistent activation can drive maladaptive fibrosis (96). Dying renal cells release damage-associated molecular patterns that recruit innate immune cells (97), which can directly injure renal cells through ROS and granule contents (98), or indirectly amplify injury by producing inflammatory mediators that recruit and activate additional immune cells (99).Ryu and colleagues identified abundant Siglec-F^+^ neutrophils in injured kidneys across unilateral ureteral obstruction, adriamycin-induced nephropathy and renal ischaemia–reperfusion injury models. This subset arose from infiltrating conventional neutrophils and was locally induced in the kidney by TGF-β1 and GM-CSF. Compared with conventional neutrophils, Siglec-F^+^ neutrophils showed greater nuclear hypersegmentation, a feature associated with distinct proteomic and functional states (100). They also produced more ROS and NETs, expressed higher levels of pro-fibrotic inflammatory mediators and showed increased COL1A1 expression. Depletion of this subset reduced collagen deposition and disease progression, whereas adoptive transfer exacerbated renal fibrosis (90). Relative to conventional neutrophils in unilateral ureteral obstruction (UUO) kidneys, Siglec-F^+^ neutrophils expressed higher surface levels of cluster of differentiation 11b (CD11b) and leucine-rich repeat-containing protein 32 (LRRC32), also known as glycoprotein A repetitions predominant (GARP), and lower levels of CD62L (100, 101). LRRC32/GARP is a cell-surface docking molecule for latent TGF-β1 and may facilitate its local activation by presenting latent TGF-β to activating mechanisms in the tissue microenvironment. Increased LRRC32/GARP expression may therefore enhance the capacity of Siglec-F^+^ neutrophils to amplify pro-fibrotic TGF-β signalling in injured kidneys, although direct evidence for this mechanism in neutrophils remains limited (102). *In vitro*, GM-CSF-pretreated bone marrow-derived neutrophils showed increased collagen type I alpha 1 chain (COL1A1) expression. These data suggest that Siglec-F^+^ neutrophils promote renal fibrosis through at least two mechanisms: activation of fibroblasts via pro-fibrotic cytokines such as TGF-β1, tumour necrosis factor (TNF) and IL-1β, and acquisition of a collagen-associated programme characterized by increased COL1A1 expression (90).

## Human translation and evidentiary standards

Human translation requires caution because neutrophil biology differs between mice and humans in lifespan, tissue distribution, marker expression and experimental accessibility (19). Neutrophil states should therefore be interpreted using integrated evidence from phenotype, transcriptomic state, tissue localisation and function, rather than by a single marker alone (103). These data suggest that Siglec-F^+^ neutrophils promote renal fibrosis primarily by amplifying fibroblast-activating inflammatory signals and by acquiring a collagen-associated programme, including increased COL1A1 expression.

In studies of human renal fibrosis, Siglec-8 expression has been examined as a possible marker of functionally convergent Siglec-expressing neutrophil states, because humans do not possess a Siglec-F gene (90). Because intratumoural fibrosis is a common feature of clear cell renal cell carcinoma (104), the authors further examined Siglec-8^+^ neutrophils in renal tissues from seven patients with this cancer. Compared with matched normal kidney tissue, tumour tissue showed more severe fibrosis and collagen deposition, and Siglec-8^+^ neutrophils were detected only in tumour components. However, caution is required: Siglec-8 is not a canonical steady-state marker of human neutrophils and is more commonly associated with eosinophils and mast cells. Because Siglec-8 is not a direct one-to-one orthologue of mouse Siglec-F, these observations should be described as evidence for a possible functionally convergent Siglec-expressing neutrophil state, rather than as evidence of a direct human equivalent of mouse Siglec-F^+^ neutrophils. Cross-species correspondence remains unresolved, and human Siglec-expressing neutrophil populations should therefore be interpreted as possible functionally convergent states requiring direct validation. Defining human-relevant neutrophil structural programmes will require lineage-aware single-cell analysis, spatial validation, proteomic confirmation, functional assays and correlation with clinical fibrotic outcomes.

In summary, the role of neutrophils in tissue repair is expanding from that of short-lived inflammatory effectors to that of structural regulators. In selected experimental settings, neutrophils have been shown to express matrix-associated programmes and contribute to local matrix organization in selected barrier tissues, temporally coordinate ECM degradation and reorganization after myocardial infarction, and acquire Siglec-F^+^ pro-fibrotic states in mouse models of cardiac and renal fibrosis. Whether these mechanisms represent shared structural programmes across organs or tissue-restricted adaptations remains unresolved. The central question is therefore no longer whether neutrophils participate in matrix remodelling, but which neutrophil subsets perform reparative or pathological structural functions, in which tissues, at which time points and under which microenvironmental cues. Resolving these questions will be essential for translating neutrophil-mediated structural immunity from a descriptive concept into targeted anti-fibrotic strategies.

## Challenges and translational agenda

Neutrophil-mediated structural immunity provides a conceptual framework for understanding failed tissue repair and its progression to fibrosis. Future studies should move beyond establishing whether neutrophils participate in matrix remodelling and instead define how distinct neutrophil states acquire reparative or pathological structural functions within specific tissues, temporal windows and microenvironmental contexts. A key unresolved question is whether these mechanisms represent shared programmes across fibrotic diseases or tissue-restricted adaptations. Findings from myocardial infarction, renal fibrosis and barrier-tissue injury models should therefore be tested in broader disease contexts, including hypertrophic scarring, pulmonary fibrosis, liver fibrosis, diabetic chronic wounds, tumour-associated stromal remodelling and vascular fibrosis.

A second priority is to determine how reparative neutrophil functions become maladaptive. During acute injury, neutrophils may support host defence, clearance of necrotic material, barrier stabilization and early provisional matrix formation. In chronic or dysregulated inflammation, however, persistent ROS production, excessive NET formation, uncontrolled degranulation and the induction or retention of pro-fibrotic neutrophil states may amplify collagen deposition, matrix stiffening and fibrotic remodelling. Causal interpretation requires particular caution. Neutrophil enrichment, activation signatures or disease-associated transcriptional states do not by themselves prove that neutrophils drive pathological remodelling. In different contexts, neutrophils may act as causal drivers, secondary responders, repair-associated bystanders or markers of injury severity. Evidence should therefore be interpreted according to experimental strength: correlative enrichment or transcriptional association is hypothesis-generating; broad neutrophil depletion can suggest functional involvement but is difficult to interpret because it can impair host defence, alter macrophage phenotypes, disrupt efferocytosis, change cytokine milieus and interfere with early repair; adoptive transfer or subset-specific depletion provides stronger evidence for state-dependent function; and pathway-specific perturbation offers more direct mechanistic support. This distinction is clinically important because broad neutrophil depletion is unlikely to be a viable therapeutic strategy. More feasible approaches should focus on selectively restraining maladaptive neutrophil programmes while preserving early reparative and antimicrobial functions.

Several methodological and translational barriers remain. It is still difficult to distinguish true neutrophil-derived matrix programmes from phagocytosed matrix material, adherent extracellular proteins or stromal contamination. Transcriptomic inference is also limited in neutrophils because their effector functions often depend on preformed granule proteins rather than newly transcribed genes. Future studies should therefore integrate lineage-aware single-cell analysis, spatial profiling, matrisome analysis, proteomic validation, *in vivo* tracing and functional perturbation. Clinically testable hypotheses include whether matrix-associated neutrophil states, the colocalisation of these states with collagen-rich niches, or persistent NET, ROS and degranulation signatures can predict fibrotic progression, scar maturation, organ dysfunction or therapeutic response.

Therapeutic translation will require precise intervention points rather than broad neutrophil depletion. Candidate strategies include limiting excessive NET formation, modulating neutrophil–matrix adhesion, interrupting pathogenic GM-CSF or TGF-β conditioning in defined contexts, regulating prolonged neutrophil survival and targeting tissue-retention signals that maintain pro-fibrotic neutrophil states. Each approach will require careful temporal and tissue-specific evaluation. Over-suppression of these programmes may impair infection control, barrier repair, inflammatory resolution or early matrix stabilization; in mechanically vulnerable tissues such as the infarcted myocardium, insufficient early matrix support may increase the risk of rupture. Thus, therapeutic strategies should aim to restrain persistent maladaptive neutrophil programmes while preserving the early reparative and antimicrobial functions required for effective tissue repair.
